# Distribution of RET Mutations and Evaluation of Treatment Approaches in Hereditary Medullary Thyroid Carcinoma in Turkey

**DOI:** 10.4274/jcrpe.2219

**Published:** 2016-03-01

**Authors:** Berna İmge Aydoğan, Bağdagül Yüksel, Mazhar Müslüm Tuna, Mehtap Navdar Başaran, Ayşen Akkurt Kocaeli, Melek Eda Ertörer, Kadriye Aydın, Sibel Güldiken, Yasin Şimşek, Züleyha Cihan Karaca, Merve Yılmaz, Müjde Aktürk, İnan Anaforoğlu, Nur Kebapçı, Cevdet Duran, Abdullah Taşlıpınar, Mustafa Kulaksızoğlu, Alptekin Gürsoy, Selçuk Dağdelen, Murat Faik Erdoğan

**Affiliations:** 1 Ankara University Faculty of Medicine, Department of Endocrinology and Metabolism, Ankara, Turkey; 2 Ankara Numune Training and Research Hospital, Clinic of Endocrinology and Metabolism, Ankara, Turkey; 3 Uludağ University Faculty of Medicine, Department of Endocrinology and Metabolism, Bursa, Turkey; 4 Başkent University Faculty of Medicine, Department of Endocrinology and Metabolism, Adana, Turkey; 5 Hacettepe University Faculty of Medicine, Department of Endocrinology and Metabolism, Ankara, Turkey; 6 Trakya University Faculty of Medicine, Department of Endocrinology and Metabolism, Edirne, Turkey; 7 Erciyes University Faculty of Medicine, Department of Endocrinology and Metabolism, Kayseri, Turkey; 8 Dokuz Eylül University Faculty of Medicine, Department of Endocrinology and Metabolism, İzmir, Turkey; 9 Gazi University Faculty of Medicine, Department of Endocrinology and Metabolism, Ankara, Turkey; 10 Trabzon Numune Training and Research Hospital, Clinic of Endocrinology and Metabolism, Trabzon, Turkey; 11 Osmangazi University Faculty of Medicine, Department of Endocrinology and Metabolism, Eskişehir, Turkey; 12 Konya Training and Research Hospital, Clinic of Endocrinology and Metabolism, Konya, Turkey; 13 Gülhane Military Medical Academy, Department of Endocrinology and Metabolism, Ankara, Turkey; 14 Necmettin Erbakan University Faculty of Medicine, Department of Endocrinology and Metabolism, Konya, Turkey; 15 Güven Hospital, Clinic of Endocrinology and Metabolism, Ankara, Turkey

**Keywords:** Sporadic medullary thyroid carcinoma, hereditary medullary thyroid carcinoma, multiple endocrine neoplasia, RET mutation

## Abstract

**Objective:**

This retrospective multicenter study, centrally conducted and supported by the Society of Endocrinology and Metabolism of Turkey, aimed to evaluate the impact of free RET proto-oncogene testing in medullary thyroid carcinoma (MTC) patients. Surgical timing, adequacy of the treatment, and frequency of prophylactic thyroidectomy (PTx) in mutation carriers were also assessed.

**Methods:**

Genetic testing for MTC and pheochromocytoma was conducted between July 2008 and January 2012 in 512 patients. Application forms and RET mutation analyses of these patients whose blood samples were sent from various centers around Turkey were assessed retrospectively. An evaluation form was sent to the physicians of the eligible 319 patients who had confirmed sporadic MTC, familial MTC (FMTC), multiple endocrine neoplasia type 2 (MEN2), or who were mutation carriers. Physicians were asked to give information about the surgical history, latest calcitonin levels, morbidity, mortality, genetic screening, and PTx among family members. Twenty-five centers responded by filling in the forms of 192 patients.

**Results:**

Among the 319 patients, RET mutation was detected in 71 (22.3%). Cys634Arg mutation was the most prevalent mutation (43.7%), followed by Val804Met in 18 patients (25.4%), and Cys634Tyr in 6 patients (8.5%). Among 192 MTC patients, the diagnosis was sporadic MTC in 146 (76.4%), FMTC in 14 (7.3%), MEN2A in 15 patients (7.9%), and MEN2B in one patient. The number of mutation carriers among 154 apparently sporadic MTC patients was 8 (5.2%). Ten patients were submitted to PTx out of twenty-four mutation carriers at a mean age of 35±19 years.

**Conclusion:**

Turkish people have a similar RET proto-oncogene mutation distribution when compared to other Mediterranean countries. Despite free RET gene testing, the number of the PTx in Turkey is limited and relatively late in the life span of the carriers. This is mainly due to patient and family incompliance and incomplete family counselling.

**WHAT IS ALREADY KNOWN ON THIS TOPIC?**

Genetic screening of germline RET mutations provides the early diagnosis of hereditary cases. Prophylactic thyroidectomy (PTx) is the only preventive option for mutation carrier family members.

**WHAT THIS STUDY ADDS?**

Turkish people has a similar RET proto-oncogene mutation distribution when compared to other Mediterranean countries i.e. Italy and France. Despite complimentary RET gene testing by the courtesy of Society of Endocrinology and Metabolism of Turkey, the number of the PTx in Turkey is limited and relatively late in the lifespan of the gene carriers. This is mainly due to patient and family incompliance and incomplete family counselling.

## INTRODUCTION

Medullary thyroid carcinoma (MTC) is a rare neuroendocrine tumor of the thyroid gland and accounts for 5% of thyroid cancers. The tumor originates from parafollicular ‘C’ cells and secretes calcitonin (CT). MTC mainly occurs sporadically (70-75%) (1). Hereditary syndromes of germline RET proto-oncogene mutations are the cause of MTC in the remaining 25-30% of patients.

The RET proto-oncogene is located on chromosome 10 (10.q11.2). It is a member of the receptor tyrosine-kinase family and comprises 21 exons. ‘C’ cells of the thyroid gland, parathyroid glands, adrenal medulla, urogenital system all express RET proto-oncogene (2). Various germline mutations of RET proto-oncogene cause distinct clinical features and influence the course of the disease (3). Multiple endocrine neoplasia type 2 (MEN 2) and familial MTC (FMTC) are both autosomal dominant inherited hereditary cancer syndromes.

MEN2A accounts for 80% of hereditary MTC syndromes; consists of MTC in all patients, pheochromocytoma in 50% and primary hyperparathyroidism in 20-30% of patients (4). Cutaneous lichen amyloidosis (10%) and Hirschprung disease (7%) may also develop in MEN2A patients (5,6). MEN2A prevalence is estimated to be 1 per 50.000 and age of diagnosis is usually 20-30 years. De novo mutations may be responsible in up to 5% of MEN2A patients (7). Codon 634 mutations are the most frequent mutations in many of European countries (8,9).

MEN2B is characterized with MTC, pheochromocytoma, mucosal neuromas, ganglioneuromatosis of the gut, and marfanoid habitus. This syndrome is caused by autosomal dominant genetic inheritance and de novo mutations equally (10). Prognosis of MEN2B is worse than MEN2A since MTC is more aggressive (11).

FMTC is classically characterized by only hereditary predisposition to MTC for at least three generations (12).

Germline mutations of the RET proto-oncogene are found in 98% of MEN2A, 95% of MEN2B, and in 88% of FMTC patients (13). Germline RET mutations can also be found in 7-10% of apparently sporadic forms of MTC by routine RET screening (14,15). Hence, only genetic testing could rule out hereditary forms of MTC.

Genetic screening of germline RET mutations is also the means for the early diagnosis of hereditary cases. Prophylactic thyroidectomy (PTx) is the only preventive option for mutation carrier family members. The association between RET mutations (genotype) and the biological behavior of tumor (phenotype) is well documented (3). Current guidelines recommend to consider risk stratification, mainly depending on the type of the mutation, in decisions on the timing of PTx (16). Age, family history, and basal/stimulated serum CT levels are the other factors which may have impact on the timing of PTx. In 2009, American Thyroid Association (ATA) recommended a 4-level risk classification for RET mutation carriers. PTx was recommended in the first year of life for MEN2B (M918T, A883F), before 5 years of age for subjects with level C (634 mutations) and also for level B (609, 611, 618, 620, 630, 631 mutations). The lower risk category (level A) involves distal codons (768, 790, 791, 804, 891). Family history of tumor behavior and family preference for these rare mutations with low genetic penetrance also need to be considered in decisions for surgery. However, there is no consensus on the management of these subjects (6,16,17,18).

This retrospective multicenter study aimed to evaluate the impact of free RET proto-oncogene testing of MTC patients. All data were evaluated in one center. The study was supported by the Society of Endocrinology and Metabolism of Turkey (SEMT). Adequacy of early diagnosis, surgical timing, and frequency of PTx in mutation carriers were also assessed.

## METHODS

Genetic testing for MTC and pheochromocytoma was conducted between July 2008 and January 2012 in 512 patients. Application forms and RET mutation analyses of these 512 patients whose blood samples were sent from various centers around Turkey were assessed retrospectively. Patients with pheochromocytoma without detected mutations and RET negative relatives of hereditary MTC patients were excluded. A total of 319 patients with familial/sporadic MTC, MEN2, and mutation carriers from known MEN2/FMTC families were found to be eligible for retrospective analysis. An evaluation form including information on the surgical history, latest CT levels, survival of the patients, and PTx had been prepared and sent to several physicians working in centers around the country. Twenty-five centers responded by filling in the forms of 192 patients.

Patients who were considered to be sporadic cases by their physicians before mutation analysis were categorized as ‘apparently sporadic’ cases. After the final analysis of the RET mutations and of the evaluation forms, the final diagnoses were categorized as MEN2A, familial/sporadic MTC, and mutation carriers from MEN2A/FMTC families.

The study was approved by the Local Ethics Committee of Ankara University Faculty of Medicine (September 5, 2012).

### RET Mutation Analysis

RET mutation analyses were performed in Düzen Laboratory Groups. Genomic DNA from patients was isolated from peripheral blood leukocytes automatically by MagNA Pure LC 2.0 (Roche Applied Science). RET gene exons 1-20 were amplified by polymerase chain reaction (PCR) using previously reported primers flanking the intron-exon junctions. PCR amplification of the exons 1-20 was carried out in a 25 µl PCR mix containing 5 ng genomic DNA, 0.1 µM of each primer, 200 µM of each dNTP, 1.5 mM MgCl, and 1.25 U Taq polymerase (19,20). PCR [touchdown (TD) TD-PCR] was carried out. TD-PCR cycling program was: initial denaturation at 95 oC for 15 min followed by 10 cycles of 1 min at 95 oC, 1 min at 62 oC with an 0.5 oC decrease of temperature per cycle and 1 min at 72 oC and an additional 25 cycles of 1 min at 95 oC, 1 min at 57 oC, 1 min at 72 oC and 10 min at 72 oC for final extension. The amplicons were analyzed in ethidium bromide-stained agarose gels and showed a single band with expected size. Then, amplicons were sequenced in an automated sequencer (ABI PRISM 3130 Genetic Analyzer; Applied Biosystems, Foster City, CA) using a BigDye Terminator v3.1 Cycle Sequencing Kit (Applied Biosystems) according to the manufacturer’s instructions. The sequence data was analyzed by using SeqScape version 2.7 (Applied Biosystems) and Sequencing Analysis version 5.1 (Applied Biosystems) softwares. The results were interpreted by a professor in medical genetics.

### Statistical Analysis

Statistical analyses were performed using Statistical Package for the Social Sciences software, version 20.0 (IBM Corp, NY, and USA). Categorical data were compared using the chi-square Fisher exact test. Group data with a normal distribution were compared using the Student t-test or analysis of variance, and nonparametric data were compared using the Mann-Whitney U or Kruskal-Wallis tests. Values were expressed as mean ± standard deviation or median as appropriate. A p-value <0.05 was considered statistically significant.

## RESULTS

**Figure 1:**
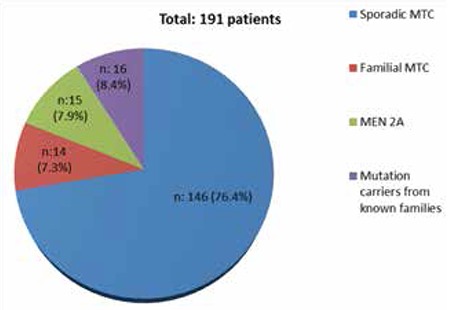
The distrubition of diagnosis for 191 medullary thyroid carcinoma patients with available clinical information (multiple endocrine neoplasia type 2 B patient was excluded). MTC: Medullary thyroid carcinoma, MEN2A: multiple endocrine neoplasia type 2 A

Figure 1, organized as a flow chart, depicts the numbers of patients included in the study at each step of the analysis. Between 2008 and 2012, among the 319 patients with hereditary/sporadic MTC whose blood samples were sent for RET analysis, mutation was detected in 71 patients (22.3%). Codon 634 mutation was detected in 39 (54.9%) patients. Cys634Arg was the most prevalent mutation (n=31) and accounted for 43.7% of all mutations. Cys634Tyr and Cys634Gly mutations were detected in 6 patients (8.5%) and in 2 patients (<1%), respectively. Val804Met germline mutation accounted for 25.4% of mutations. Distribution of RET mutations is summarized in Table 1.

Among the 192 MTC patients with available clinical information, 146 patients had sporadic MTC, 15 patients MEN2A, 14 patients FMTC, and one subject had MEN2B. Sixteen patients were mutation carriers from known families. However, so-called FMTC patients may not fulfill the diagnostic criteria yet since three generations of follow-up is mandatory (12).

Distribution of RET proto-oncogene mutation in 191 patients with available clinical information is summarized in Table 2, and distribution of RET mutations according to final diagnosis is summarized in Table 3. One MEN2B patient with Met918Thr mutation was excluded before statistical analyses.

RET mutation was detected in 8 of 154 patients (5.2%) defined as ‘apparently sporadic’ cases. Among these patients, three had Cys634Arg, three Val804Met, one Cys618Ser, and one subject had Y790Phe mutation.

Mean age at diagnosis was similar in the two genders (44.2±14 years in females vs. 44.6±13.6 years in males, p=0.7), but was significantly lower in the patients diagnosed with genetic testing compared to those diagnosed with classical clinical features (31±18.2 years vs. 46.5±12.7 years, p<0.01). Mean age at diagnosis was also not statistically different between sporadic MTCs (47.6±1.05 years) and FMTCs (41.3±2.9 years) (p>0.05). MEN2A patients were younger (35.6±2.78 years) than sporadic and FMTCs at presentation (p<0.01) (a summary of preoperative clinical data is given in Table 4).

Mean follow-up period was 40±27 months in the whole group. The stage of tumor at diagnosis and preoperative CT levels are summarized in Table 4. Preoperative CT level was significantly higher in sporadic cases compared with the MEN2A and FMTCs (p<0.05) (Table 4).

Follow-up data was available for 138 patients. On their most recent follow-up visit, 80 patients (58%) were in full remission, 21 patients (15%) had locoregional disease (17 operable, 4 inoperable) and 29 (21%) had distant metastases. Seven sporadic and one MEN2A patient (6%) died due to distant metastases.

Sorafenib was the most preferred chemotherapeutic drug in metastatic patients (n=14). Conventional chemotherapeutics were chosen for 9 metastatic patients.

The mean number of surgical procedures was not statistically different between sporadic (1.67±1.05) and hereditary cases (1.31±1.5) (p=0.09).

A significant number of mutation carriers (n=14) had not been operated yet although RET mutation carrier status was known at least for a year. Three out of fourteen subjects had elevated basal CT levels at the time of the study (Table 5). PTx or total thyroidectomy with central lymph node dissection was performed only in 10 out of 24 patients (41.6%). Mean age at thyroidectomy was 35±19 (12-60) years. Our study population included only five pediatric cases, and three of them had not undergone PTx although they had Codon 634 mutation.

## DISCUSSION

This retrospective multicenter study aimed to evaluate the impact of complimentary RET proto-oncogene testing in MTC patients. The timing of the surgical intervention, adequacy of diagnosis, treatment in familial/sporadic MTCs, and frequency of PTx for mutation carriers were also assessed.

More than 145 germline mutations of RET proto-oncogene have been identified during the past 20 years, and it has been shown that common mutations are localized in eight exons (exons 5, 8, 10, 11, 13, 14, 15, 16) (21). Previously, the approach for the screening of family members of this autosomal dominant inherited disorder consisted of repeated analyses of basal and/or stimulated CT measurements. In 1993, two different groups identified RET proto-oncogene mutations as the cause of hereditary cases and MEN2 syndromes, and RET genetic testing has since become the method of screening gradually (22,23). The prognosis of MTC is not favorable compared to differentiated thyroid carcinomas. Tumor stage at the time of diagnosis is the most important prognostic factor (24). Curability of metastatic disease is quite difficult. When possible, early diagnosis and treatment is the cornerstone of the management.

In asymptomatic carriers, mutation analysis facilitates early diagnosis and treatment of the disease. The ATA guidelines recommend the use of a four-level risk classification for RET mutations (16). Risk categories are important to decide the timing of PTx for mutation carriers. Although the biological behavior of the disease is usually comparable within the families, it can occasionally be variable within the same family (25,26).

It is well-known that early PTx reduces cancer mortality to lower than 5% in MEN2A patients (27). The primary goal of early prophylactic surgery is to prevent lymph node metastases. Unfortunately, cervical lymph node metastases are found in 70% of patients when the nodule becomes palpable (28). On the other hand, surgery at early ages is associated with increased morbidity (29). Thus, annual follow-up of stimulated CT has been suggested to guide the timing and extent of surgery. Elisei et al (30) achieved cure in RET mutation carriers who were treated early after stimulated CT levels were detected to be elevated. However, in a recent study, it has been reported that basal and stimulated CT levels failed to detect MTC before surgery in three of the 31 (10%) mutation carriers (31). European Multiple Endocrine Neoplasia (EUROMEN) study also demonstrated that 75% (12/16) of codon 634 mutations developed MTC before the age of five. Thus, early diagnosis with RET proto-oncogene testing, risk stratification, and stimulated CT levels are the mainstay for the timing of PTx in hereditary cases.

Our results in this retrospective analysis showed that, although free genetic testing is available with the courtesy of SEMT and conducted centrally by the society itself, the number of PTx is still low in Turkey and the timing of surgical intervention is late. Current data showed that prophylactic/total thyroidectomy was performed only in 10 patients out of 24 mutation carriers identified and mean age of hereditary cases at surgery was relatively late [35±19 years (12-60)]. Fourteen of the known mutation carriers had not yet undergone surgery at the time of the study due to patient and family incompliance and/or incomplete family counselling. Unfortunately, people deny genetic diseases, parents still have hesitations about surgical interventions for their children, especially during younger ages, and healthcare professionals may not be able to overcome these problems.

MEN2A is frequently caused by the mutations in codons 634, 620, 618, 611, 609. Mutations at codon 634 of exon 11 account for 85% of cases and approximately half of them are cysteine to arginine amino acid substitution (Cys634Arg) (32). In FMTC, more than 85% of patients have mutations in exons 10 and 11 (22). Other rare mutations were described in exon 13 (codons 768, 790, 791), exon 14 (codons 804,844) and exon 15 (codon 891).

This is the largest mutation analysis ever performed in Turkish population and most common RET proto-oncogene mutation reported was codon 634 mutation (54.9%). Val804 mutation was also an important mutation, accounting for 25.4% of RET mutations in Turkish population, especially in FMTC patients (79%) although some of these patients did not fulfill the diagnostic criteria yet. Codon 804 mutation is considered to be a low risk in ATA 2009 guidelines, and individual-, patient-, and family-based management is suggested. In our series, 11 patients with FMTC had Val804Met mutation and they underwent surgery at a mean age of 47.2±10.9. Two patients had local recurrence, one had metastatic disease, and despite relatively late age of surgery, the remaining eight subjects were in remission. Surgical technique could of course be questioned in the local recurrence. Thus, our results are concordant with ATA guidelines, where distal codons (768, 790, 791, 804, 891) were categorized as lower risk mutations with low genetic penetrance.

EUROMEN multicenter study showed that the most prevalent RET mutation in the European population was Cys634 with 67.6% frequency (6). The multicenter ItaMEN network analysis demonstrated that codon 634 at exon 11 was the most affected codon (34.8%) followed by Val804Met mutation (19.6%) among Italian MEN2 syndromes (33). Val804 mutations were threefold frequent in Italian and French when compared to German families. These data are concordant with the results in our series since Val804Met mutations are also prevalent in Turkey (25.4%). Sánchez et al (34) reported that the most frequent RET mutation in MEN2A Spanish families was C634Y, occurring in 73% (22/30) of cases and this finding was attributed to founder effect. A recent German study also demonstrated that RET mutations were distributed fairly similarly among German, French, and Italian families. The mutations in codon 634 were the most prevalent (36%), and codon 790 mutations were also frequent in Germany compared to Italy and France (13% vs. 4% and 4%) (35). In our series, codon 790 mutations were very rare, accounting for 1.4% of all mutations. Other studies from China, India, and Korea also demonstrated that Codon 634 mutations accounted for 60-80 % of hereditary cases. Interestingly, Val 804 mutation was not reported in China (36) and Korea (37) and had a lower frequency in a small Indian study (38) compared to current and other European studies (7% vs. 25.4%) (33,35).

Another important finding in accordance with previous findings of our group and of the others is that genetic testing revealed eight mutation carriers (hereditary cases) (5.2%) among 154 clinically diagnosed as sporadic MTCs (13,14). This data underlines the necessity of genetic screening for all MTC patients (15,16). On the other hand, frequency of mutation carriers among apparently sporadic cases was lower than in the previous TURKMEN study (10.7%), presumably due to increased awareness and genetic testing among physicians in our country. Current guidelines recommend genetic testing for all MTC cases and if positive, clinical screening for pheochromocytoma and primary hyperparathyroidism (16,21). In the rare situation when the clinical criteria for hereditary syndromes are present but RET mutation analyses are negative, clinical screening of siblings is also recommended. As a cost-effective approach, ATA and ETA recommend to test MEN2-specific exons (i.e. exons 10,11) principally and if found negative, continue testing for exons 13, 14, and 15 subsequently (16,21).

The mean number of surgical procedures were higher in our sporadic cases compared to familial cases, but no statistically significant difference was observed. These findings may reflect delayed timing of diagnosis and small number of PTx for hereditary cases.

This study shows that the Turkish people has a similar RET proto-oncogene mutation distribution when compared to other Mediterranean countries such as Italy and France. Despite complementary RET gene testing made possible by the courtesy of SEMT, the number of the PTx in Turkey is limited and the surgical intervention is performed at a relatively late stage in the lifespan of the gene carriers. This is mainly due to patient and family incompliance and incomplete family counselling. Healthcare professionals seem to be unsuccessful to overcome these problems for the moment. Physicians and health authorities should be aware of this situation. Legal measures could be considered for the families who refuse healthcare for their children.

## Figures and Tables

**Table 1:**
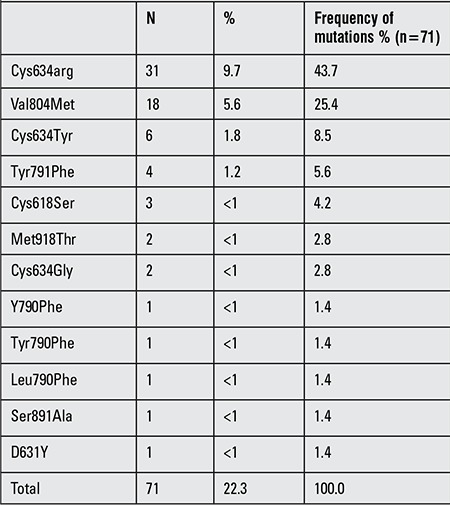
Distribution of RET proto-oncogene mutations in 71 out of 319 patients with hereditary sporadic medullary thyroid carcinoma or pheochromocytoma who had been tested for RET proto-oncogene

**Table 2:**
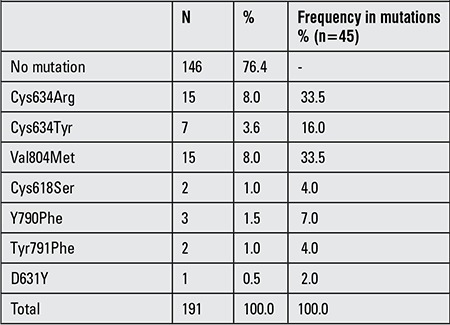
Distribution of results of the RET proto-oncogene testing in 191 patients with available clinical information

**Table 3:**
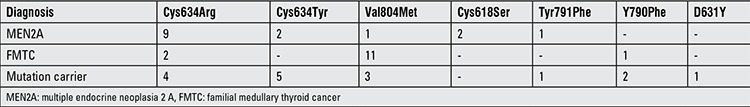
Distribution of RET mutations according to final diagnosis

**Table 4:**
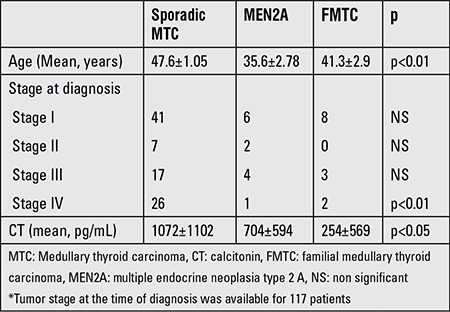
Preoperative clinical data of patients*

**Table 5:**
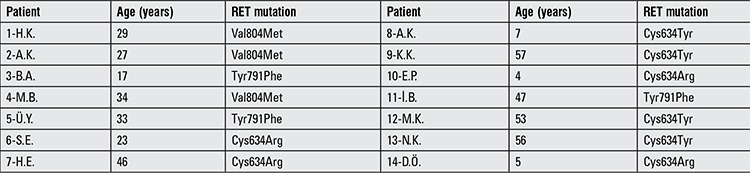
Mutations and age of the patients with known RET mutation who had not yet undergone thyroid surgery

## References

[1] Marsh DJ, Learoyd DL, Robinson BG (1995). Medullary thyroid carcinoma: recent advances and management update. Thyroid.

[2] Machens A, Lorenz K, Dralle H (2009). Constitutive RET tyrosine kinase activation in hereditary medullary thyroid cancer: clinical opportunities. J Intern Med.

[3] Eng C, Clayton D, Schuffenecker I, Lenoir G, Cote G, Gagel RF, Amstel HK, Lips CJ, Nishisho I, Takai SI, Marsh DJ, Robinson BG, Frank-Raue K, Raue F, Xue F, Noll WW, Romei C, Pacini F, Fink M, Niederle B, Zedenius J, Nordenskjöld M, Komminoth P, Hendy GN, Mulligan LM, et al (1996). The relationship between specific RET proto-oncogene mutations and disease phenotype in multiple endocrine neoplasia type 2. International RET mutation consortium analysis. JAMA.

[4] Raue F, Frank-Raue K, Grauer A (1994). Multiple endocrine neoplasia type 2. Clinical features and screening. Endocrinol Metab Clin North Am.

[5] Gagel RF, Levy ML, Donovan DT, Alford BR, Wheeler T, Tschen JA (1989). Multiple endocrine neoplasia type 2a associated with cutaneous lichen amyloidosis. Ann Intern Med.

[6] Frank-Raue K, Rybicki LA, Erlic Z, Schweizer H, Winter A, Milos I, Toledo SP, Toledo RA, Tavares MR, Alevizaki M, Mian C, Siggelkow H, Hüfner M, Wohllk N, Opocher G, Dvořáková S, Bendlova B, Czetwertynska M, Skasko E, Barontini M, Sanso G, Vorländer C, Maia AL, Patocs A, Links TP, Groot JW, Kerstens MN, Valk GD, Miehle K, Musholt TJ, Biarnes J, Damjanovic S, Muresan M, Wüster C, Fassnacht M, Peczkowska M, Fauth C, Golcher H, Walter MA, Pichl J, Raue F, Eng C, Neumann HP, International RET Exon 10 Consortium (2011). Risk profiles and penetrance estimations in multiple endocrine neoplasia type 2A caused by germline RET mutations located in exon 10. Hum Mutat.

[7] Schuffenecker I, Ginet N, Goldgar D, Eng C, Chambe B, Boneu A, Houdent C, Pallo D, Schlumberger M, Thivolet C, Lenoir GM (1997). Prevalence and parental origin of de novo RET mutations in multiple endocrine neoplasia type 2A and familial medullary thyroid carcinoma. Le Groupe d’Etude des Tumeurs a Calcitonine. Am J Hum Genet.

[8] Machens A, Niccoli-Sire P, Hoegel J, Frank-Raue K, Vroonhoven TJ, Roeher HD, Wahl RA, Lamesch P, Raue F, Conte-Devolx B, Dralle H, European Multiple Endocrine Neoplasia (EUROMEN) Study Group (2003). European Multiple Endocrine Neoplasia (EUROMEN) Study Group. Early malignant progression of hereditary medullary thyroid cancer. N Engl J Med.

[9] Niccoli-Sire P, Murat A, Rohmer V, Franc S, Chabrier G, Baldet L, Maes B, Savagner F, Giraud S, Bezieau S, Kottler ML, Morange S, Conte-Devolx B, French Calcitonin Tumors Group (GETC) (2001). Familial medullary thyroid carcinoma with noncysteine RET mutations: phenotype-genotype relationship in a large series of patients. J Clin Endocrinol Metab.

[10] Carlson KM, Bracamontes J, Jackson CE, Clark R, Lacroix A, Wells SA, Goodfellow PJ (1994). Parent-of-origin effects in multiple endocrine neoplasia type 2B. Am J Hum Genet.

[11] Robbins J, Merino MJ, Boice JD, Ron E, Ain KB, Alexander HR, Norton JA, Reynolds J (1991). Thyroid cancer: a lethal endocrine neoplasm. Ann Intern Med.

[12] Brandi ML, Gagel RF, Angeli A, Bilezikian JP, Beck-Peccoz P, Bordi C, Conte-Devolx B, Falchetti A, Gheri RG, Libroia A, Lips CJ, Lombardi G, Mannelli M, Pacini F, Ponder BA, Raue F, Skogseid B, Tamburrano G, Thakker RV, Thompson NW, Tomassetti P, Tonelli F, Wells SA, Marx SJ (2001). Guidelines for diagnosis and therapy of MEN type 1 and type 2. J Clin Endocrinol Metab.

[13] Eng C (1999). RET proto-oncogene in the development of human cancer. J Clin Oncol.

[14] Elisei R, Romei C, Cosci B, Agate L, Bottici V, Molinaro E, Sculli M, Miccoli P, Basolo F, Grasso L, Pacini F, Pinchera A (2007). RET genetic screening in patients with medullary thyroid cancer and their relatives: experience with 807 individuals at one center. J Clin Endocrinol Metab.

[15] Erdogan MF, Gürsoy A, Ozgen G, Cakir M, Bayram F, Ersoy R, Algün E, Cetinarslan B, Cömlekçi A, Kadioglu P, Balci MK, Yetkin I, Kabalak T, Erdogan G (2005). RET proto-oncogene mutations in apparently sporadic Turkish medullary thyroid carcinoma patients: Turkmen study. J Endocrinol Invest.

[16] Kloos RT, Eng C, Evans DB, Francis GL, Gagel RF, Gharib H, Moley JF, Pacini F, Ringel MD, Schlumberger M, American Thyroid Association Guidelines Task Force (2009). Wells SA Jr. Thyroid.

[17] Rich TA, Feng L, Busaidy N, Cote GJ, Gagel RF, Hu M, Jimenez C, Lee JE, Perrier N, Sherman SI, Waguespack SG, Ying A, Grubbs E (2014). Prevalence by age and predictors of medullary thyroid cancer in patients with lower risk germline RET proto-oncogene mutations. Thyroid.

[18] Grubbs EG, Waguespack SG, Rich TA, Xing Y, Ying AK, Evans DB, Lee JE, Perrier ND (2010). Do the recent American Thyroid Association (ATA) Guidelines accurately guide the timing of prophylactic thyroidectomy in MEN2A?. Surgery.

[19] Zhou Y, Zhao Y, Cui B, Gu L, Zhu S, Li J, Liu J, Yin M, Zhao T, Yin Z, Yu C, Chen C, Wang L, Xiao B, Hong J, Zhang Y, Tang Z, Wang S, Li X, Ning G (2007). RET proto-oncogene mutations are restricted to codons 634 and 918 in mainland Chinese families with MEN2A and MEN2B. Clin Endocrinol (Oxf).

[20] Hofstra RM, Landsvater RM, Ceccherini I, Stulp RP, Stelwagen T, Luo Y, Pasini B, Höppener JW, Amstel HK, Romeo G, et al (1994). A mutation in the RET proto-oncogene associated with multiple endocrine neoplasia type 2B and sporadic medullary thyroid carcinoma. Nature.

[21] Elisei R, Alevizaki M, Conte-Devolx B, Frank-Raue K, Leite V, Williams GR (2013). 2012 European thyroid association guidelines for genetic testing and its clinical consequences in medullary thyroid cancer. Eur Thyroid J.

[22] Mulligan LM, Kwok JB, Healey CS, Elsdon MJ, Eng C, Gardner E, Love DR, Mole SE, Moore JK, Papi L, et al (1993). Germ-line mutations of the RET proto-oncogene in multiple endocrine neoplasia type 2A. Nature.

[23] Donis-Keller H, Dou S, Chi D, Carlson KM, Toshima K, Lairmore TC, Howe JR, Moley JF, Goodfellow P, Wells SA (1993). Mutations in the RET proto-oncogene are associated with MEN 2A and FMTC. Hum Mol Genet.

[24] Frank-Raue K, Rondot S, Raue F (2010;30). Molecular genetics and phenomics of RET mutations: Impact on prognosis of MTC. Mol Cell Endocrinol.

[25] Waguespack SG, Rich TA, Perrier ND, Jimenez C, Cote GJ (2011). Cote. Management of medullary thyroid carcinoma and MEN2 syndromes in childhood. Nat Rev Endocrinol.

[26] Lombardo F, Baudin E, Chiefari E, Arturi F, Bardet S, Caillou B, Conte C, Dallapiccola B, Giuffrida D, Bidart JM, Schlumberger M, Filetti S (2002). Familial medullary thyroid carcinoma: clinical variability and low aggressiveness associated with RET mutation at codon 804. J Clin Endocrinol Metab.

[27] Gagel RF, Tashjian AH, Cummings T, Papathanasopoulos N, Kaplan MM, DeLellis RA, Wolfe HJ, Reichlin S (1988). The clinical outcome of prospective screening for multiple endocrine neoplasia type 2a. An 18-year experience. N Engl J Med.

[28] Moley JF, DeBenedetti MK (1999). Patterns of nodal metastases in palpable medullary thyroid carcinoma: recommendations for extent of node dissection. Ann Surg.

[29] Kahraman T, Groot JW, Rouwe C, Hofstra RM, Links TP, Sij-mons RH, Plukker JT (2003). Acceptable age for prophylactic surgery in children with multiple endocrine neoplasia type 2a. Eur J Surg Oncol.

[30] Elisei R, Romei C, Renzini G, Bottici V, Cosci B, Molinaro E, Agate L, Cappagli V, Miccoli P, Berti P, Faviana P, Ugolini C, Basolo F, Vitti P, Pinchera A (2012). The timing of total thyroidectomy in RET gene mutation carriers could be personalized and safely planned on the basis of serum calcitonin: 18 years experience at one single center. J Clin Endocrinol Metab.

[31] Pelizzo MR, Torresan F, Boschin IM, Nacamulli D, Pennelli G, Barollo S, Rubello D, Mian C (2013). Early, prophylactic thyroidectomy in hereditary medullary thyroid carcinoma: A 26-year monoinstitutional experience. Am J Clin Oncol.

[32] Figlioli G, Landi S, Romei C, Elisei R, Gemignani F (2013). Medullary thyroid carcinoma (MTC) and RET proto-oncogene: mutation spectrum in the familial cases and a meta-analysis of studies on the sporadic form. Mutat Res.

[33] Romei C, Mariotti S, Fugazzola L, Taccaliti A, Pacini F, Opocher G, Mian C, Castellano M, degli Uberti E, Ceccherini I, Cremonini N, Seregni E, Orlandi F, Ferolla P, Puxeddu E, Giorgino F, Colao A, Loli P, Bondi F, Cosci B, Bottici V, Cappai A, Pinna G, Persani L, Verga U, Boscaro M, Castagna MG, Cappelli C, Zatelli MC, Faggiano A, Francia G, Brandi ML, Falchetti A, Pinchera A, Elisei R, ItaMEN network (2010). Multiple endocrine neoplasia type 2 syndromes (MEN 2): results from the ItaMEN network analysis on the prevalence of different genotypes and phenotypes. Eur J Endocrinol.

[34] Sánchez B, Robledo M, Biarnes J, Sáez ME, Volpini V, Benítez J, Navarro E, Ruiz A, Antiñolo G, Borrego S (1999). High prevalence of the C634Y mutation in the RET proto-oncogene in MEN 2A families in Spain. J Med Genet.

[35] Machens A, Lorenz K, Sekulla C, Höppner W, Frank-Raue K, Raue F, Dralle H (2013;15). Molecular epidemiology of multiple endocrine neoplasia 2: implications for RET screening in the new millenium. Eur J Endocrinol.

[36] Qi XP, Chen XL, Ma JM, Du ZF, Fei J, Yang CP, Cheng J, Song QZ, Han JS, Jin HY, Chen ZG, Wang JQ, Yang YP, Ying RB, Liu WT, Zhao Y, Chen CY, Jiang HL, Ke HP, Zhang XN (2012). RET proto-oncogene genetic screening of families with multiple endocrine neoplasia type 2 optimizes diagnostic and clinical management in China. Thyroid.

[37] Chung YJ, Kim HH, Kim HJ, Min YK, Lee MS, Lee MK, Kim KW, Ki CS, Kim JW, Chung JH (2004). RET proto-oncogene mutations are restricted to codon 634 and 618 in Korean families with multiple endocrine neoplasia 2A. Thyroid.

[38] Sharma BP, Saranath D (2011). RET gene mutations and polymorphisms in medullary thyroid carcinomas in Indian patients. J Biosci.

